# RNAs and RNA-Binding Proteins in Immuno-Metabolic Homeostasis and Diseases

**DOI:** 10.3389/fcvm.2019.00106

**Published:** 2019-08-20

**Authors:** Esam S. B. Salem, Andrew D. Vonberg, Vishnupriya J. Borra, Rupinder K. Gill, Takahisa Nakamura

**Affiliations:** ^1^Division of Endocrinology, Cincinnati Children's Hospital Medical Center, Cincinnati, OH, United States; ^2^Department of Pharmacology and Systems Physiology, University of Cincinnati College of Medicine, Cincinnati, OH, United States; ^3^Division of Developmental Biology, Cincinnati Children's Hospital Medical Center, Cincinnati, OH, United States; ^4^Department of Pediatrics, University of Cincinnati College of Medicine, Cincinnati, OH, United States; ^5^Department of Metabolic Bioregulation, Institute of Development, Aging and Cancer, Tohoku University, Sendai, Japan

**Keywords:** obesity, RNA methylation, PKR, snoRNA, CCR4-NOT complex, RNA silencing, Ago2, TRBP

## Abstract

The increasing prevalence of worldwide obesity has emerged as a major risk factor for type 2 diabetes (T2D), hepatosteatosis, and cardiovascular disease. Accumulating evidence indicates that obesity has strong inflammatory underpinnings tightly linked to the development of metabolic diseases. However, the molecular mechanisms by which obesity induces aberrant inflammation associated with metabolic diseases are not yet clearly defined. Recently, RNAs have emerged as important regulators of stress responses and metabolism. RNAs are subject to changes in modification status, higher-order structure, and cellular localization; all of which could affect the affinity for RNA-binding proteins (RBPs) and thereby modify the RNA-RBP networks. Proper regulation and management of RNA characteristics are fundamental to cellular and organismal homeostasis, as well as paramount to health. Identification of multiple single nucleotide polymorphisms (SNPs) within loci of fat mass- and obesity-associated protein (FTO) gene, an RNA demethylase, through genome-wide association studies (GWAS) of T2D, and functional assessments of FTO in mice, support the concept that disruption in RNA modifications leads to the development of human diseases including obesity and metabolic disorder. In obesity, dynamic alterations in modification and localization of RNAs appear to modulate the RNA-RBP networks and activate proinflammatory RBPs, such as double-stranded RNA (dsRNA)-dependent protein kinase (PKR), Toll-like receptor (TLR) 3 and TLR7, and RNA silencing machinery. These changes induce aberrant inflammation and the development of metabolic diseases. This review will describe the current understanding of the underlying causes of these common and altered characteristics of RNA-RBP networks which will pave the way for developing novel approaches to tackle the pandemic issue of obesity.

## Introduction

In the past several decades, dramatic and rapid changes in lifestyle and dietary trends have led to obesity becoming a worldwide problem, bringing with it a host of chronic metabolic diseases, including type 2 diabetes (T2D), non-alcoholic steatohepatitis (NASH), non-alcoholic fatty liver disease (NAFLD), and cardiovascular disease (CVD). The global prevalence has reached epidemic proportions. In 2015, ~603.7 million adults and 107.7 million children were considered obese, and yet these figures are predicted to continue to increase worldwide (1). Despite the enormous burden on global public health, effective preventive and/or therapeutic strategies are limited, as mechanisms underlying this cluster of pathologies remain unclear.

In 2007, two independent genome-wide association studies (GWAS) evaluating patients with T2D revealed fat mass- and obesity-associated protein (FTO) as the first GWAS-identified diabetes susceptibility locus (2, 3). Initial reports suggested multiple single nucleotide polymorphisms (SNPs) within the first intron of FTO found on chromosome 16 were significantly associated with obesity in humans (4), although the association between FTO SNPs and FTO expression has been controversial (5–8). Clarity was provided in mouse model studies in which it was demonstrated that FTO plays a vitally important role in the regulation of fat mass, adipogenesis, and body weight (9). Importantly, FTO is the first *N*6-methyladenosine (m6A) demethylase of eukaryotic messenger RNA (mRNA) (10, 11), and the regulation of adipogenesis is associated with demethylase activity of FTO (12, 13). Subsequently, reports indicate that FTO demethylates not only m6A, but also *N*6,2′-*O*-dimethyladenosine (m6Am), *N1*-methyladenosine (m1A), and regulate RNA stability (14, 15), with findings that further support the concept of RNA modification's role in the pathogenesis of obesity.

The biological significance of RNA modification remains largely unknown in metabolic disorders, yet accumulated evidence suggests that the dynamic regulation of RNA modifications could have profound impacts on mRNA splicing, translation, maturation of non-coding microRNAs (miRNAs), and interactions with RNA-binding proteins (16, 17). Recent findings, however, indicate that disruption of RNA homeostasis is often associated with cellular and metabolically-driven stresses that trigger inflammatory responses (18, 19). In obesity, a wide range of inflammatory and stress conditions are often generated in liver and/or adipose tissue, among other insulin-targeted tissues. These Inflammatory responses occur, at least in part, through activation of inflammatory RNA-binding proteins (RBPs), which includes double-stranded RNA (dsRNA)-dependent protein kinase (PKR) (20), Toll-like receptor (TLR) 3 and TLR7 (21–23). These responses result in chronic, low grade, local inflammation capable of disrupting systemic metabolic homeostasis (19, 24, 25). In addition, functional alterations of RBPs involved in RNA metabolism are associated with disruption in cellular energy homeostasis (26, 27). Therefore, altered RNA networks associated with RNA modification as a consequence of obesity may be a crucial step causing the inflammatory signaling cascades and abnormal RNA metabolism that lead to metabolic disorders. This review will summarize the advances and discoveries in the field of RNA modifications and RBPs, and discuss their contributions toward the pathogenesis of obesity and associated sequelae.

### Discovery of Fat Mass- and Obesity-Associated Gene, FTO

The GWAS studies identified strong signals within intron 1 of the FTO gene associated with an increase in T2D, body mass index (BMI), and obesity (Table 1). Approximately 16% of subjects homozygous for the risk allele of FTO weighed at minimum 3 kg more than subjects lacking the allele (54), with a 31% increased risk of developing obesity as compared to those subjects containing the non-risk allele (29). Two independent analyses in French and Sardinian studies confirmed a significant association of SNPs in the first intron of the FTO gene with increased BMI in individuals of European origin (4, 5). Moreover, these studies identified three additional SNPs within intron 1 of the FTO gene that were associated with severe childhood and morbid adult obesity (rs1421085 and rs17817449) (4, 5), and with obesity-related traits (rs9930506) (45), thus yielding a population attributable risk of 22% for common obesity (55). Subsequent studies reported that polymorphisms in the *FTO* genetic locus are also associated with onset and development of insulin resistance, metabolic syndrome, atherosclerosis, systemic hypertension, and alteration in C-reactive protein levels (Table 1) (56–60). The effects vary among different ethnic populations, thereby correlating the higher susceptibility of certain ethnicities to obesity with other modifying factors (61).

**Table 1:** SNPs in FTO locus.

**FTO *SNP*s**	**Phenotype observed**	**Region or country of population studied**	**Number of studies**	**Total enrollment across all studies, (*n* = subjects + healthy controls)**	**References**
rs9939609 (Intron 1)	Correlations with increased BMI, obesity, and in a few divergent studies increased risk for metabolic diseases (ex: T2D or insulin sensitivity), though a few studies reported no direct correlation for T2D. Some studies also showed that diet or exercise can attenuate some of the effects or heightened risk of SNP presence	Denmark, Finland, Europe, Germany, Brazil, Spain, United States, Japan, Netherlands, South Asia, Asia, Africa, China, Portugal	18	518,014	(28–44)
rs9930506 (Intron 1)	Correlation with BMI, hip circumference and total body weight in Sardinian patients and validated using 3,467 GenNet samples	Sardinia	1	6,148	(45)
rs8050136 (Intron 1)	Correlation with BMI and obesity, but not with insulin sensitivity, and second study found FTO SNP does not affect weight loss post-pregnancy	Germany and United States	2	1,700	(46, 47)
rs17818902 (Intron 1)	No direct correlation between BMI or obesity to specific SNP	Czech Republic	1	107	(48)
rs17817449 (Intron 1)	SNP correlates with 22% increased risk of obesity in adults and children	Europe	1	8,000	(5)
rs1558902 (Intron 1)	SNP does not affect weight loss following obesity	United States	1	742	(49)
rs1421085 (Intron 1)	Increased risk for obesity and/or T2D with SNP presence, but physical activity can attenuate effects	Europe and Global	2	22,585	(50, 51)
rs1121980 (Intron 1)	Increased risk for obesity and higher BMI with SNP presence, but physical activity can attenuate effects	Europe	2	131,795	(52, 53)

*The association between SNPs of fat mass- and obesity-associated (FTO) gene resulting in an impact on the development of obesity-associated phenotypes in multi-ethnic populations in different countries*.

In a study investigating the link between the obesity-associated FTO SNPs and FTO expression, expression quantitative trait locus (eQTL) analysis failed to demonstrate that SNPs affected FTO expression in human tissues (6, 62, 63). Charting the cis-regulatory circuitry within the FTO locus by the circular chromosome conformation capture (4C) method revealed that the locus physically interacts with the promoter sequence required for expression of Iroquois homeobox protein 3 (IRX3), which plays an early role during neural development, located approximately 500 kilobases (kb) away (64). In the human brain, obesity-associated FTO SNPs correlated with higher expression of IRX3, but not FTO, indicating that increased expression of IRX3 may be due to the genetic variation within FTO being responsible for the obesity-associated phenotypes. Consistently, IRX3-deficient mice are leaner and do not develop obesity after being place on a high fat diet, and hypothalamus-specific dominant-negative IRX3 mice also present with the same phenotype as IRX3-deficient mice (64). An independent study also reported that a FTO SNP, rs1421085, disrupts a conserved motif of the regulatory gene AT-rich interaction domain 5B (ARID5B), causing induction of IRX3 and IRX5 expression levels (65). This disruption leads to an autonomous developmental shift from beige energy-producing to white energy-storing adipocytes, which can explain the obesity-associated phenotypes observed in FTO risk variant(s).

These findings suggest the obesity and obesity-associated phenotypes observed in individuals who have FTO SNPs in intron 1 may be attributed to their potential to influence expression of IRX3 and IRX5, instead of FTO itself (66, 67). Nonetheless, animal models investigating FTO gene functions have clearly shown that FTO regulates energy metabolism, body weight, and adipogenesis (68). FTO-deficient mice exhibit significant reductions in fat and lean body mass due to greater energy expenditure and over-activation of the sympathetic system, suggesting the involvement of FTO in the regulation of energy homeostasis (69). Conversely, transgenic mice carrying extra copies of FTO intake significantly more food with greater weight gain, enhanced fat accumulation in adipose tissues, and impairment of glucose tolerance in a copy number-dependent manner (70). These metabolic phenotypes are consistent with the expression pattern of FTO mRNA abundant within cerebral tissues, including the hypothalamic centers, and points to FTO possibly controlling appetite and energy balance (71–73), although mice with FTO depletion within the hypothalamus presented with only a slight reduction in their body weight as compared to controls (72). Overall, these series of findings suggest that FTO plays a critical role regulating body weight and metabolism.

### The Role of FTO in RNA Demethylation

**Figure 1:**
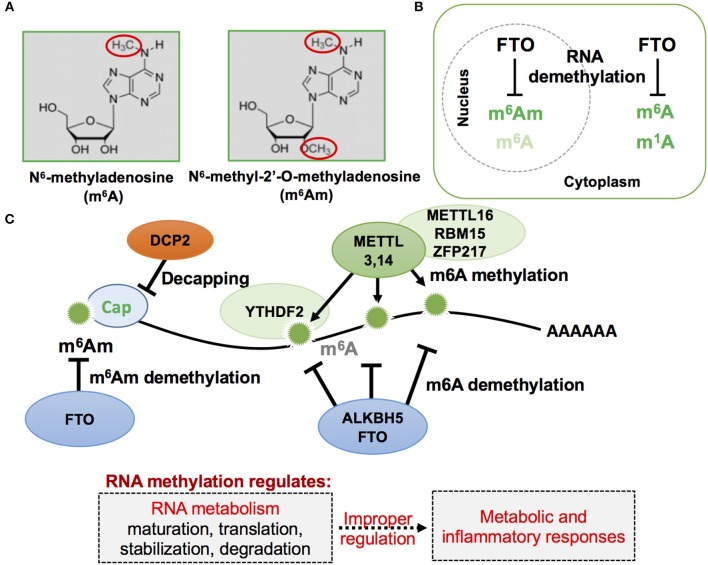
Schematic representation of mRNA methylation modification in eukaryotic cells. **(A)** RNA methylation in adenosine often occurs, and has been previously reported, by attacking nitrogen and oxygen atoms as indicated in red circles of the following two described nucleotides: m^6^A and m^6^Am. **(B)** Cellular localization of FTO, m^6^A, m^6^Am, and m^1^A. FTO, first described as a nuclear protein, was later found to exist in both the cell nucleus and cytoplasm when observed in different mammalian cell lines. **(C)** RNA m^6^A and m^6^Am methylation and demethylation on mRNA. The mRNA methylation status affects mRNA metabolism and its improper regulation could result in abnormal metabolic and inflammatory responses.

FTO contains within its sequence a Fe^2+^ binding motif and a 2-oxyglutarate (2OG)-dependent oxygenase (71). Structurally, FTO protein is closely related to the bacterial DNA demethylase Alpha-ketoglutarate-dependent dioxygenase (AlkB) and the mammalian AlkB homologs ABH1 and ABH2, which act on nucleic acids such as DNA and transfer RNA (tRNA) (74–77). A subsequent study identified FTO as the N6-methyladenosine (m^6^A) demethylase of mRNA (Figure 1) (78). In mammals, adenosine methylation can directly be reversed by at least two demethylases, FTO and AlkB homolog 5 (ALKBH5) (79), while methyltransferase like 3 and 14 (METTL3,14) are enzymes that mediate the catalytic conversion of adenosine to m6A (80, 81). Recently, m6A has been found to play important roles in inflammation and antiviral responses (82). Genome-wide transcriptome profiling following depletion of ALKBH5 was able to identify differentially expressed genes that regulate the immune response to viral infections, with METTL14 specific depletion altering pathways important in controlling metabolic reprogramming, stress responses, and aging (83). Furthermore, depletion of METTL3 and METTL14 has also been shown to lead to elevated levels of type I interferon responses to myriad viral infections (83, 84).

Of note, a recent study identified a novel role of FTO for RNA modification of the first encoded nucleotide next to the 7-methylguanosine cap, N6,2-prime-O-dimethyladenosine (m6Am), in controlling mRNA stability (Figure 1). This RNA modification is the preferred substrate of FTO, rather than m6A (15, 85), and selective demethylation of m6Am through FTO appears to destabilize mRNA (86). Analysis of m6Am transcriptomes revealed that its transcripts are more stable when compared to mRNAs starting from different nucleotides. The enhanced stability of m6Am-initiated transcripts appears to correlate with resistance by these transcripts to DCP2, an mRNA-capping enzyme. A more recent study confirmed that FTO could effectively demethylate both m6A and cap m6Am and identified additional RNA substrates of FTO, including m1A in tRNA, m6A in U6 RNA, and internal and cap m6Am in small nuclear RNAs (snRNAs) (14). While FTO-mediated demethylation of internal m6A is compellingly associated with a plethora of biological processes, including, but not limited to, adipogenesis and inflammatory responses (11, 12, 71, 87), more comprehensive investigation of FTO-mediated demethylation action on other RNA species in response to environmental cues may provide deeper insights into cellular energy metabolism. For instance, RNA methylation-mediated regulation of mRNA stability could affect the efficiency of protein translation that accounts for a majority of cellular energy expenditure (27, 88).

Beyond simply the importance of FTO-mediated RNA methylation lies the presence of effective RBPs capable of recognizing and binding these methylated RNAs. One such RBP, YTH N6-Methyladenosine RNA Binding Protein 2 (YTHDF2), is an RNA-binding protein that belongs to the YTH domain-containing family. It regulates decay, destabilization, and degradation of methylated genes by selectively binding to previously described m6A-methylated mRNA within the G(m6A)CU/A consensus, or by recruiting CCR4-NOT deadenylase complex (see the CCR4-NOT section below) within P-bodies found in the cytoplasm (89–92). YTHDF2 is implicated as having crucial roles in metabolic and inflammatory processes via modulation of expression levels of targeted and methylated mRNA transcripts. For example, FTO depletion inhibits adipogenesis by prolonging the cell cycle during adipocyte differentiation via decreased expression of methylated cyclin A2 (CCNA2) and cyclin-dependent kinase 2 (CDK2). This occurs through the action of m6A reader YTHDF2, thereby indicating the important role of YTHDF2 in FTO-mediated adipogenic control (93). In addition, depletion of YTHDF2 significantly enhanced the stimulation of multiple inflammatory signaling pathways such as of NF-κB and mitogen-activated protein kinases (MAPKs), and elevated the expression levels of inflammatory cytokines such as TNF-α and IL6 (94). The consensus from these studies points to the role of m6A and its binding proteins in the regulation of immune-metabolic homeostasis and disorders such as obesity.

Technical advances have enhanced our ability to define the distribution profile and dynamic properties of m6A methylation by using sensitive and specific approaches. While chemical and antibody-based approaches have been developed and provided us important findings, there appear to be some limitations in these approaches. For instance, methylated RNA immunoprecipitation with anti-methylated RNA-specific antibodies and sequencing (MeRIP-seq) has been used to detect global m6A levels within 100 nucleotides length (95, 96). However, these approaches make identification and quantification of specific m6A methylation levels highly variable due to relatively poor sensitivity and weak specificity at single-nucleotide resolutions (97, 98). Furthermore, these antibody-based techniques make quantifying m6A stoichiometry difficult, especially when evaluating the deposition and level of m6A methylation fraction of modified transcripts (98–102). To overcome these difficulties, a recent study has developed a new antibody-independent method, the M6A-SensiTivE RNA digestion and sequencing (MASTER-seq) (103), which is based on the ability of the MazF RNA endonuclease to cleave RNA specifically at unmethylated sites within “ACA” motifs (104). The MASTER-seq enables the detection and quantification of m6A at the single-base resolution at about 16–25% of all methylation sites. This study indicates that MASTER-seq could be used as a novel resource to quantify m6A levels in systemic scales of different cell types and compartments, as well as in varying pathologic conditions such as obesity (103).

### Molecular Pathways Sensing Ribose 2′-O-Methylation in Innate Immunity and Metabolism

**Figure 2:**
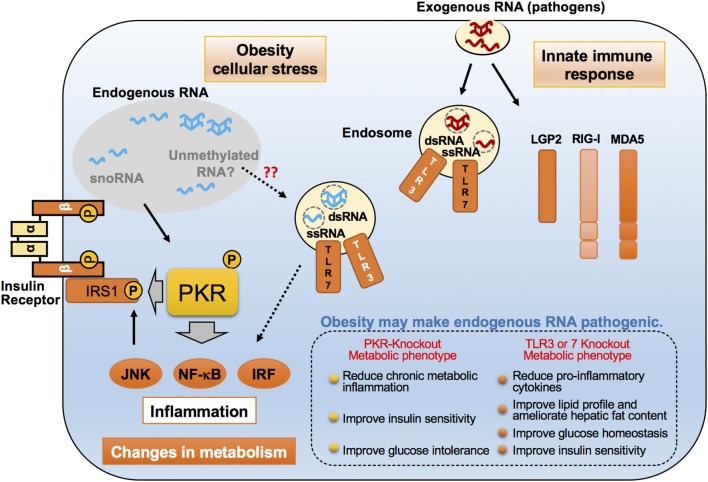
Emerging roles of endogenous RNAs in inflammatory responses in obesity. Proposed molecular mechanisms for pro-inflammatory dsRNA-binding proteins such as PKR, TLR3, and TLR7, in recognizing exogenous and endogenous dsRNAs. These dsRNA-binding proteins distinguish and interact with virus-derived exogenous RNAs upon viral infection, and lead to the induction of an immune response. However, under cell stress conditions, such as saturated fatty acid exposure, these proteins are believed to sense changes in endogenous dsRNA networks through their interactions, thereby resulting in chronic inflammation that alters the metabolic state. These dsRNA-binding proteins accomplish this by phosphorylating the inhibitory serine found on insulin receptor substrate-1 (IRS-1), a critical insulin signaling component.

While m6A and m6Am appear to highly be associated with metabolic and inflammatory regulation, there are more than 100 different modes of RNA modifications, some of which are also involved in the innate immune defense mechanism in host cells (105, 106). Ribose 2′-O-methylation (2′-O-methylation) is one such RNA modification. In eukaryotes, mRNAs usually carry a 5′-cap structure that is methylated on the first and oftentimes the second cap-proximal nucleotides (N1 and N2, where N corresponds to any nucleotide), resulting in Cap1- (m7GpppNmN) or Cap2- (m7GpppNmNm) mRNA. These methylations serve as a molecular signature of “self” to escape detection by innate immunity, and therefore mRNAs that do not contain any 2′-*O*-methylation close to the cap are marked as “non-self” and trigger the release of type I IFNs. Cap-specific mRNA (nucleoside-2′-O-)-methyltransferase 1 (CMTR1) dictates mRNA cap1 2′-O-ribose methylation of the mRNA 5′-cap structure. Importantly, dysregulation in CMTR1 expression is associated with several pathologies related to self-recognition immune mechanism from non-self RNAs. Consistently, it has been shown that uncapped viral RNAs can trigger an interferon-mediated antiviral response from the host. Mechanistically, several pro-inflammatory RBPs, including RIG-I and MDA5, are potent regulators that bind and discriminate between “self” and “non-self” RNA (Figure 2). For instance, 2′-O-methylation of the 5′-cap structure abolishes interaction with RIG-I and MDA5, leading to the inability to recognize signaling of non-self RNA by the innate immune system. Conversely, virally transcribed RNA outside the nucleus generally exhibits a 5′ triphosphate, which can be recognized by RIG-I as “non-self” RNA.

TLR7, similarly to TLR3, is an inflammatory RBP and involved in the recognition of 2′-O-methylation status of RNA and causes the induction of inflammatory responses (Figure 2). Initial studies examining the role of RNA modifications in immune responses revealed that the base modifications, including 2′-O-methylation at the ribose moiety on *in vitro* transcripts, prevented TLR activation. Conversely, an investigation into the role of RNA modifications in naturally occurring bacterial tRNA species demonstrated that tRNAs can induce the secretion of IFN-α in plasmacytoid dendritic cells (pDCs) through TLR7 signaling in a methylation status-dependent manner (107–111). Importantly, studies in humans and mouse models have implicated pathways sensing these “non-self” RNA in obesity-induced inflammation. Mice lacking TLR7 exhibited an attenuation in metabolic inflammation and hyperglycemia on HFD (112). In addition, TLR7-deficient mice were protected from diet-induced atherosclerosis due to a reduction of atherosclerotic lesion inflammatory cytokines and systemic levels of serum amyloid A (113).

The expression level of TLR7 has been consistently very low in healthy human arterial specimens and higher in human atherosclerotic lesions, suggesting its involvement in atherosclerosis (114). Other studies have similarly reported that higher expression of TLR7 in atherosclerotic plaques and carotid arteries atheroma in human patients (115, 116). In the pathogenesis of metabolic diseases, a series of studies implicated endogenous RNA behaving like a pathogen which activated TLR7 through changes in the status of RNA modification. Recent studies identified minimal trinucleotide motifs within a 9-mer oligoribonucleotide that are capable TLR7 antagonists (117). Development of specific inhibitors of TLR7-sensing 2′-O-methylated RNAs may facilitate future therapeutic strategies to manage complex immunometabolic disorders such as obesity and atherosclerosis.

### Diverse Roles of Small Nucleolar RNAs (snoRNAs) Involved in 2′-O-Methylation

**Figure 3:**
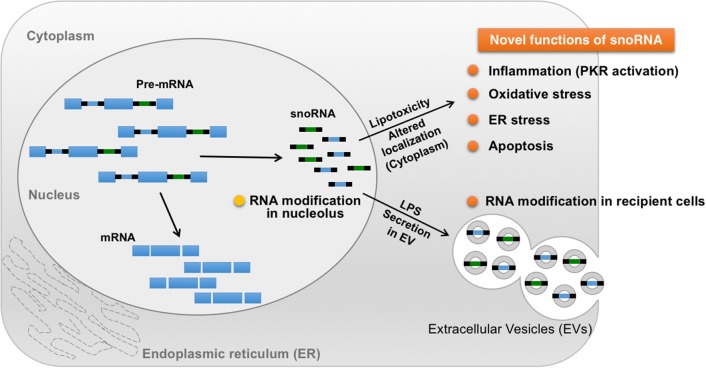
Novel roles of snoRNA in response to metabolic and inflammatory stresses. A majority of snoRNAs are generated from intronic sequences during the splicing process. Under metabolically-driven stress conditions, levels of cytoplasmic snoRNAs are upregulated, thereby inducing oxidative stress, ER stress, and even cell death. In addition, LPS-treatment in macrophages enhances secretion of snoRNAs carried by extracellular vesicles (EV), leading to snoRNA-mediated RNA modification in distant EV recipient cells, which could contribute to the pathogenesis of systemic metabolic and inflammatory disorders in obesity.

Small nucleolar RNAs (snoRNAs) are small non-coding RNAs that manage chemical modifications of other RNAs including ribosomal RNA (rRNA) and tRNA (118). SnoRNAs are stratified into two main groups according to their specific structural features: C/D box and H/ACA box (119). The majority of these snoRNAs localize in the nucleolus and canonically target nascent rRNAs for 2′-O-methylation (Figure 3) (106, 120). In addition, snoRNAs help regulate alternative splicing that leads to the creation of small RNA fragments that have microRNA (miRNA)-like activity (121–123). Intriguingly, snoRNAs are involved in mediating the lipotoxic effects in metabolically-driven stress responses (Figure 3). When cells are overloaded by pro-inflammatory saturated long chain fatty acids such as myristate, palmitate or stearate, snoRNAs [box C/D snoRNAs U32a (SNORD32a), U33 (SNORD33), U34 (SNORD34), and U35a (SNORD 35a)], located within introns 2, 4, and 6 of the targeted *Rpl13a* gene, are induced transcriptionally or by pre-mRNA stabilization in the cytoplasm, but not in the nucleoli. Inactivation of these snoRNAs by antisense oligonucleotides protects cells against palmitate-induced reactive oxygen species (ROS) production, ER stress, and subsequent cell death, independent of 2′-O-methylation of rRNA targets. A subsequent genome-wide shRNA-based screen recently identified nuclear export factor 3 (NXF3) as a transporter that can change the nucleo-cytoplasmic distribution pattern of these box C/D snoRNAs. Of note, these snoRNAs are involved in glucose intolerance and cholesterol trafficking mechanisms after treatment with fatty acids (120). For instance, knockdown of the *Rpl13a s*noRNAs U32a, U33, U34, and U35a increased glucose-mediated insulin secretion in mice and systemic glucose tolerance (124), whereas snoRNA U60 (SNORD60) contributed to intracellular cholesterol trafficking, independent of its predicted function of methylating ribosomal RNAs (125). Interestingly, inflammation stimulates secretion of the *Rpl13a* snoRNAs from cultured macrophages through extracellular vesicles and these secreted snoRNAs travel in the circulation to functionally regulate 2′-O-methylation in distant tissues (Figure 3) (126), although it remains unclear whether these secreted snoRNAs trigger inflammatory responses in the recipient cells. These findings uncover a previously unappreciated role of snoRNAs linking intra- and inter-cellular signaling to systemic metabolic and inflammatory regulations. Additionally, microdeletions encompassing the *SNORD116* cluster on the paternal chromosome 15q11.2 are associated with Prader-Willi syndrome (PWS), a syndrome in which affected individuals displaying profound food-seeking behavior and are, consequently, severely obese with or without diabetes (127). A recent animal study demonstrated that hypothalamic loss of *SNORD116* recapitulates the hyperphagia of PWS (128). Although the molecular mechanism remains unresolved, these findings also suggest diverse roles of snoRNAs in the pathogenesis of obesity.

### PKR Interactions With Endogenous RNAs in Inflammatory Responses and Protein Translation

Several RBPs, including TLR7, function as receptors for pathogen-associated molecular patterns (PAMPs) and danger-associated molecular patterns (DAMPs) capable of inducing atypical inflammation under obese conditions (19, 129–131). PKR, also known as eukaryotic initiation factor 2-alpha kinase 2 (EIF2AK2), is another such sensor projected to be an adept regulator of innate immunity and downstream protein translation, against pathogen infection in higher eukaryotes (Figure 2) (132). PKR, together with PERK, HRI, and GCN2, is a member of the eIF2α kinases and able to suppress protein translation through phosphorylation of eIF2α, which results in inhibition of guanine nucleotide exchange activity of the eIF2B subunit (133, 134). PKR can also phosphorylate several other substrates, including inhibitor κB (IκB), and stimulate multiple inflammatory signaling pathways such as c-Jun N-terminal kinase (JNK), MAPKs, and nuclear factor κB (NF-κB) (135). The functions of PKR have been well-documented during viral infection, particularly RNA viruses, in which virus-derived dsRNAs are recognized by PKR and bound to its N-terminal two dsRNA-binding domains (dsRBDs), resulting in activation of the c-terminal intramolecular kinase domain (135). Importantly, these inflammatory and protein translational events are implicated in the pathogenesis of obesity, as demonstrated by activation of JNK, NF-κB, and phosphorylation of eIF2 α, which are all induced in metabolic tissues, including liver and white adipose tissue, of obese mice and in humans (136). With these unique intrinsic molecular functions of PKR, it was perhaps not surprising that in obesity, PKR was found to be activated in metabolic tissues, and, upon activation, PKR induces JNK activation and eIF2α phosphorylation, followed by the inhibitory serine phosphorylation of insulin receptor substrate-1 (IRS-1), a critical insulin signaling component, by directly acting on IRS-1 or by indirectly activating JNK respectively (20). PKR activation was also observed in metabolic tissues of obese humans and, after bariatric surgery, PKR activation was reduced, followed by decreases in JNK and inhibitor of kappa β kinase (IKK β) activation, as well as IRS-1 phosphorylation found within subcutaneous adipose tissue biopsied from these patients (137).

Intriguingly, PKR activation is induced by cellular stressors, including palmitate-induced lipotoxicity and endoplasmic reticulum (ER) stress, in the absence of pathogenic infection. The PKR activation induced by these cellular stresses, when compared to the robust activation by dsRNA or analogs like polyinosinic-polycytidylic acid (Poly(I:C)), are generally not drastically increased, but are sufficient to induce JNK activation and eIF2α phosphorylation (20). These findings raise the possibility that PKR may ably sense nutrients or other cellular molecules, including endogenous RNA species, under metabolic stress conditions. Since PKR's defined RNA-binding activity is necessary for functional activation, a PKR RNA-binding defect variant (PKR-RM), which contains a point mutation at the lysine 64 residue, lost its ability to respond to palmitate, implicating a critical role of PKR's RNA-binding activity with endogenous dsRNAs during palmitate-induced PKR activation (20). Furthermore, immunoprecipitation (RIP)-seq analysis, wherein the authors of the study immunoprecipitated both PKR-RM and -WT, followed by purification and sequence analysis of bound RNAs, revealed that snoRNAs were in fact the majority of enriched RNAs that interacted with PKR-WT (Figure 3) (138). Importantly, H/ACA box snoRNAs contain a common secondary structure made up of two hairpins and two single-stranded regions, termed a hairpin-hinge-hairpin-tail structure, and this structure contains a dsRNA-like region (139). Furthermore, the important contribution of snoRNAs to PKR activation and regulation of metabolism was discerned by observing that palmitate exposure selectively interacted with PKR-WT only, and not with PKR-RM. Consistently, another study found that snoRNAs also can bind and activate PKR *in vivo* when overexpressed in cells (138).

In addition to interacting with snoRNAs, PKR can be activated by sensing endogenous RNAs, including inverted Alu repeats (IRAlus), mitochondrial tRNAs, and mRNAs, in mouse and human cells (140, 141). For instance, 5′-UTR of IFN-γ mRNA mediates specific activation of PKR, leading to inhibition of IFN-γ translation through a negative-feedback mechanism (142, 143), which could validate how the innate immune system over-activates upon sensing stress stimuli and then incrementally ameliorate the inflammatory effects of excessive IFN-γ levels. Tumor necrosis factor alpha (TNF-α), TNF-α 3′-UTR mRNA can also bind and activate PKR both *in vivo* and *in vitro* (144). Intriguingly, activation of PKR enables TNF-α mRNA splicing without blocking translation for efficient expression of TNF-α (145). These findings also implicate the physiological relevance of PKR activation in the inflammatory response to endogenous RNA species.

### PKR-Deficient Mouse Models in Obesity

**Figure 4:**
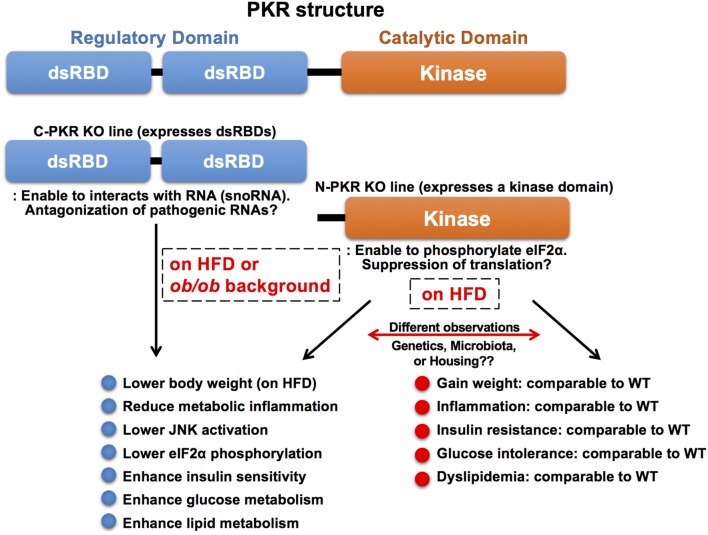
Molecular structure and knockout mouse models of PKR. PKR is a serine/threonine kinase composed of a N-terminal regulatory domain containing two dsRBDs and a C-terminal catalytic (kinase) domain. The two dsRBDs bind to dsRNA leading to conformational change and kinase activation. There are two PKR-deficient mouse models used in the study of PKR function. Both PKR-deficient mouse models are incomplete knockouts and retain partial functionality of PKR. The N-PKR KO model lacks two dsRBDs, but expresses a kinase domain that enables the phosphorylation of eIF2α. Conversely, the C-PKR KO model lacks the kinase domain, but expresses two dsRBDs. C-PKR KO mice showed beneficial metabolic phenotypes in the pathogenesis of obesity, however, there were contradictory findings in metabolic phenotypes observed in N-PKR KO mice. The differences may be due to environmental, genetic, and/or epigenetic factors that arise within different N-PKR KO mouse colonies.

Although PKR appears to play a critical role in immuno-metabolic regulation and, indeed, several studies have shown that PKR-deficiency in mice exhibits beneficial immuno-metabolic phenotypes, contradicting observations have been reported (20, 146–149). These observations may be attributed, at least in part, to the use of different PKR-deficient mouse lines and their corresponding husbandry environment. The most widely used PKR-deficient mouse models carry a deletion in either the N terminal (N-PKR KO) or C terminal domain of PKR (C-PKR KO), resulting in expression of truncated forms of PKR (Figure 4). Specifically, both PKR-deficient mouse models are incomplete knockouts and retain partial functionality of PKR (150–152). The N-PKR KO model lacks two dsRBDs, but expresses a kinase domain that enables the phosphorylation of eIF2α. Conversely the C-PKR KO model lacks the kinase domain but express the two dsRBDs. Differences in signaling responses to specific stimuli have been observed in cells isolated from C-PKR KO or N-PKR KO mice.

Interestingly, the C-PKR KO mice on a mixed 129Sv x BALB/c background in a HFD study gained less weight and exhibited improved glucose tolerance and insulin sensitivity with decreased JNK activation and eIF2α phosphorylation within metabolic tissues as compared to wild-type control mice (20). In addition, C-PKR KO mice were protected from intralipid-induced acute inflammation and insulin resistance (20). Subsequently, these mice were backcrossed to C57Bl/6 background, then crossed with genetically obese leptin-deficient mice (*ob/ob*) to generate C-PKR KO-*ob/ob* mice. The beneficial metabolic phenotypes, including improved systemic glucose tolerance and insulin sensitivity, were also observed in C-PKR KO-*ob/ob* mice, while body weight was not affected (147). This combination of findings demonstrate that PKR plays a role as a critical regulator of metabolically-driven stresses and inflammation in obesity, making it a potential target for obesity-associated metabolic and inflammatory conditions.

In contrast, there are contradictory reports in metabolic phenotypes of N-PKR KO mice bred on a C57Bl/6 background. One study analyzing N-PKR KO mice in obesity validated the findings that N-PKR KO mice were protected from developing HFD-induced insulin resistance and glucose intolerance, due to a reduction in c-Jun N-terminal kinase activity which resulted in improved insulin signaling within insulin-sensitive tissues (146). Conversely, another study reported that N-PKR KO mice did not show such protective phenotypes on HFD and there was only a mild influence on adipose tissue inflammation in N-PKR KO mice (148). This study also reported no significant difference in inflammation levels when comparing WT and N-PKR KO macrophages after treatment with palmitic acid. Similarly, an independent study showed that N-PKR KO mice on HFD showed no significant differences in body weight or glucose levels compared to their control mice, although insulin levels were markedly decreased (149).

In another example of differences between the PKR-deficient mouse lines, N-PKR KO mice displayed severe disease-like of symptoms upon dextran sodium sulfate (DSS)-induced colitis (153), whereas C-PKR KO mice showed reduced sensitivity to DSS-induced colitis (154). These differences could be attributed to genetic/epigenetic differences, which may arise due to variations in independently maintained PKR KO mouse colonies, or possible differences in the gut microbiome (155). More importantly, it is known that cells in N-PKR KO mice still carry a kinase domain that enables the phosphorylation of PKR substrates including eIF2α (151). As the kinase lacks the two regulatory dsRNA domains, the kinase activity is likely to be unregulated *in vivo*, and its substrates can be varied in different cells and under myriad cellular conditions. Of note, intact PKR has been observed to regulate intestinal inflammation and appears to be indispensable for DSS-induced colitis. These findings implicate a critical role of PKR in the gut microenvironment's immune function, which could systemically affect humoral immune responses and activate signaling pathways and molecules, including the kinase domain of N-PKR KO mice.

In the C-PKR KO mice, maintaining expression of dsRBDs, in addition to deletion of the kinase domain, of PKR may be associated with beneficial effects during the pathogenesis of obesity. The dsRBDs can still interact with endogenous RNAs, including snoRNAs, particularly under metabolically-driven stress conditions (138, 151). Interaction of these PKR dsRBDs with RNAs in the absence of a kinase domain may result in neutralizing endogenous “pathological” RNAs such as the cytoplasmic snoRNAs triggering stress responses. It is noteworthy in several reports that treatment of PKR inhibitors, which generally target the kinase activity, show beneficial impacts on metabolism and diseases (156–160). Similar to the phenotypes observed in C-PKR KO mice, TLR3 deficiency, again a sensor of dsRNA associated with viral infections that stimulate innate immunity, improves glucose tolerance and reduces liver steatosis in obese mice maintained in specific pathogen-free conditions (21). Concurrent findings in PKR and TLR3 support the possibility that endogenous RNA species become “pathological” and are recognized by the dsRNA sensors, leading to the deterioration of systemic glucose and energy metabolism in obesity.

### Regulatory Machinery of miRNAs in Immuno-Metabolism

Hypothetically, if nutrient and pathogen response systems are integrated, then involvement of pathogen sensors would be anticipated during metabolic regulation and stimulate the pursuit toward understanding the potential role of PKR in metabolism (19). Other than PKR, components of RNA-induced silencing complex (RISC) and RISC-loading complex (RLC) might assume such an integrative role, as recent evidence indicates that the host miRNA pathway represents just such an adaptive antiviral defense mechanism (161, 162). In addition, it has become obvious that regulatory mechanisms governed by miRNAs are vital for glucose maintenance and lipid homeostasis in metabolic tissues (163, 164). Mammalian Dicer, a main component of RLC, interacts with two closely related dsRNA binding proteins, TRBP (TAR RNA binding protein) and PACT (Protein activator of PKR), both of which regulate Dicer stability and contribute to RLC formation (165, 166). In addition, both molecules interact with PKR and are involved in inflammatory responses through regulation of PKR kinase activity and downstream activation of JNK, eIF2α, and IKK (147).

**Figure 5:**
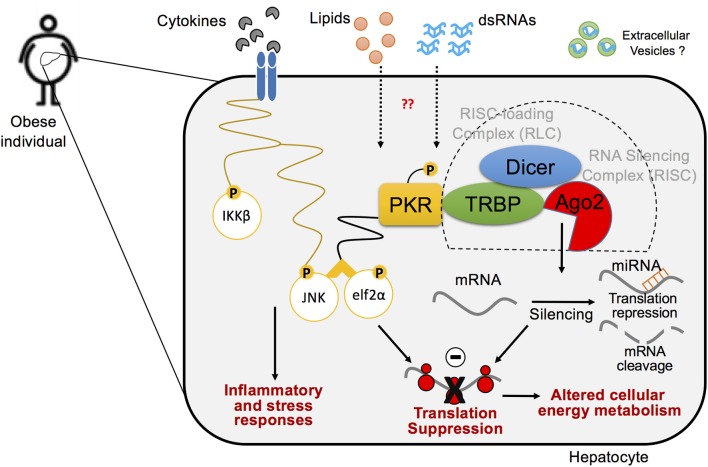
A cross-talk between inflammatory pathways and RNA silencing machinery in hepatocytes in the pathogenesis of obesity. Biological interaction between inflammatory signaling pathway and protein translation regulation via PKR within the hepatocyte in metabolic stress responses. PKR is activated by lipid-mediated stress within obese metabolic tissues and induces concordant inflammatory responses, in which endogenous dsRNAs mediate PKR activation. Upon activation, PKR forms an inflammatory protein complex, using TRBP, Dicer, and RNA-induced silencing complex (RISC), which leads to protein translation suppression via phosphorylation of elf2α and enhances inflammation via activation of JNK pathway. Ago2-mediated RNA silencing that regulates protein translation also plays a critical role in disputing metabolic homeostasis in the pathogenesis of obesity.

Intriguingly, the formation of a PKR complex featuring TRBP and Dicer is enhanced in metabolically-driven stress conditions and in the obese liver, where TRBP phosphorylation induced by MAPKs, such as JNK, contribute to the stabilization of Dicer and its interaction with PKR (Figure 5) (147, 167). Phosphorylation levels of TRBP are induced in obese livers when PKR is activated, and inactivation of hepatic TRBP in *ob/ob* obese mice results in a significant reduction of JNK activity and eIF2α phosphorylation, which coincides with the alleviation of systemic glucose intolerance, insulin resistance, and hepatic steatosis (147). Similar regulations were observed in cells undergoing palmitate-induced metabolic stress conditions. While there is still controversy surrounding the role of TRBP in regulating PKR activity (168, 169), these findings suggest that, in the setting of metabolic inflammation, MAPK-induced TRBP phosphorylation leads to PKR activation, followed by eIF2α phosphorylation and JNK activation, as well as increased global miRNA expression, which are all observed simultaneously in obese liver.

### Role of RISC in Metabolic Regulation in Obese Liver

TRBP is involved in glucose metabolism, at least in part, by regulating inflammatory responses in the obese liver, though questions remain as to whether another important function of TRBP regulating miRNA biogenesis and RNA silencing is involved in metabolic regulation. Recently, the vital role of hepatic miRNA-mediated events have been demonstrated in the pathogenesis of obesity and associated diseases (170). Global dysregulation of miRNA expression triggers in obese livers of humans and mice leads to the induction of the vast majority of miRNAs (171, 172), consistent with increased TRBP phosphorylation associated with enhanced Dicer stability and function. In addition, hepatic *Dicer*-deficient mice, lacking a gene encoding a central enzyme for miRNA generation, were characterized phenotypically as presenting with systemic metabolic disorders, hepatic lipid accumulation, and tumor development (173, 174). Therefore, it is reasonable and important to clarify the role of RISC in obesity-induced pathophysiology.

In the liver, Ago1 and Ago2 are prominent members of the Argonaute family and the main components of RISC that carry out RNA silencing (175). Hepatic Ago1 appears to be unnecessary for obesity-induced pathophysiology, as it has been reported that deletion of hepatic Ago1 had no significant effect on weight gain, glucose tolerance, or insulin sensitivity under obese conditions (27). Conversely, hepatic Ago2-deficiency significantly aided and improved glucose metabolism, even in conditions of insulin receptor antagonist treatment, HFD challenge, and deletion of hepatic 5′-AMP-activated protein kinase catalytic subunit alpha-1 (AMPKα1) which regulates energy metabolism (27). Ago2 has been shown to regulate the expression of a number of specific miRNAs including miR-802, miR-103/107, and miR-148a/152, which deteriorate glucose and lipid metabolism after being generated by Ago2's unique “slicer” activity (27, 171, 176–178). In addition, Ago2-deficient hepatocytes are characterized as having enhanced mitochondrial oxidation and ATP consumption, which appears to be associated with less weight gain and enhanced energy expenditure observed in hepatic Ago2-deficient mice (27). An independent study also demonstrated that hepatic Ago2-deletion from *ob/ob* mice led to reduced body weight with lowered basal and fasted blood glucose levels, as well as improved insulin sensitivity (179). Hepatic Ago2-deficiency resulted in increased expression of *AMPK*α*1* with activation of AMPK, a critical regulator of energy metabolism, reduced expression of miR-148a, and enhanced general protein translation that contributed to ATP consumption and AMPK activation (27, 179). These findings suggest that Ago2 has the unique function of regulating energy production and consumption in the liver, and these data point to hepatic Ago2-mediated RNA silencing being a key regulator of energy metabolism under obesity-related metabolic conditions.

Of note, the distribution of RNA modifications was enriched in the untranslated regions of mRNAs and near Argonaute protein binding regions, which indicates a potential role of RNA modification in RNA silencing (180). For instance, it was previously reported that the binding sites for the Argonaute proteins overlap within the 3 UTRs of the m6A residues (181). In addition, a significant number of 5-methylcytosine (m5C) candidate sites are located within mRNA containing binding regions for the Argonaute proteins (182). These results demand further study to determine the interconnected role of RNA modification and Argonaute proteins toward metabolic regulation, as changes in these modifications occurring in obesity may impact the post-transcriptional gene regulation via Ago2-mediated RNA silencing.

### Metabolic Regulation Through the CCR4-NOT Complex

**Figure 6:**
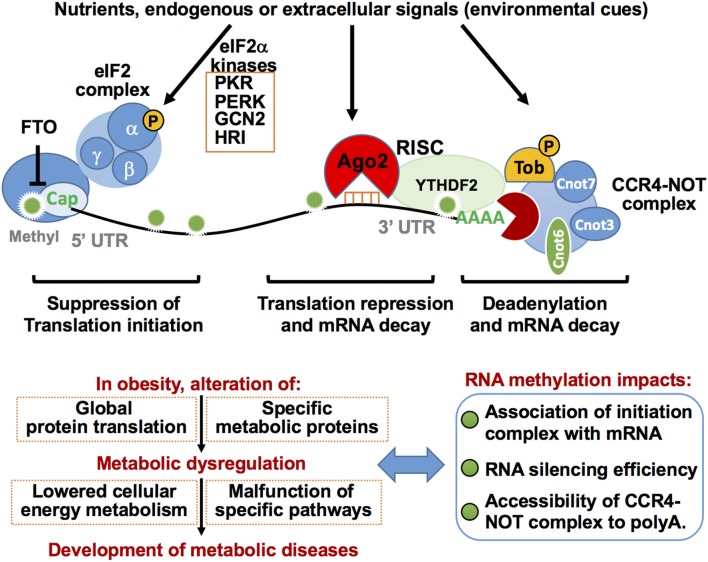
Post-transcriptional regulation of mRNA translation repression eIF2a complex, Ago2-mediated RNA silencing, and decay and deadenylation via CCR4-NOT complex in metabolic regulation. RNA stability is increased by m^7^Gppp cap-adjacent m^6^Am. The m^7^Gppp cap is recognized by the protein initiation complex for protein translation. Conversely, eIF2a phosphorylation induced by eIF2a kinases in response to a variety of environmental cues results in inhibition of protein translation. Removal of the m^7^Gppp cap by the decapping enzyme DCP2 is a key step in mRNA decay. Ago2-mediated RNA silencing represses mRNA translation and induces its decay. The CCR4-NOT protein complex binds and deadenylates the poly(A) tail of target mRNA to stimulate mRNA decay. Ago2 RNA silencing machinery interacts with CCR4-NOT complex resulting in rapid mRNA decay and/or translation repression. TOB protein recruits CCR4-NOT deadenylase to a specific mRNA poly(A) tail by direct binding to Cnot7. Functions of these components are highly integrated into cellular metabolic homeostasis, at least in part, by regulating the efficiency of global protein translation and/or expression of specific metabolic proteins. Altered RNA methylation patterns on mRNA induced in obesity may affect these processes and thereby disrupt cellular metabolic homeostasis.

A poly(A) sequence at the 3′ end of mRNA tail contributes to post-transcriptional gene regulation by acting on mRNA metabolic stability and frequency of translation (183). Shortening of poly(A) tails by deadenylation induces mRNA decay from 3′ end (184). The major deadenylase in eukaryotic cells is the CCR4-NOT complex, which is composed of Cnot1-Cnot3, Cnot6, Cnot6L, and Cnot7-Cnot11 (Figure 6) (185–187). Furthermore, the CCR4–NOT complex is involved in post-transcriptional silencing via interactions with RISC (188, 189). Importantly, the CCR4–NOT complex is implicated during development of metabolic diseases such as obesity, diabetes, liver steatosis, and energy metabolism by metabolizing mRNAs of energy metabolic-associated genes and thus reducing their expression levels through deadenylase activity (190). Mice haplodeficient in Cnot3 that were fed a HFD, or in an *ob/ob* background, exhibited reduced hepatic and adipose fat deposition, improved glucose tolerance, and enhanced insulin sensitivity (190). Mechanistically, in these Cnot3 haplodeficient conditions, expression levels of pyruvate dehydrogenase kinase 4 (*Pdk4*) and insulin-like growth factor binding protein 1 (*Igfbp1*) mRNAs were higher with longer poly(A) tails when compared to controls due to reduced deadenylation activity (190). Liver-specific Cnot3 conditional deletion resulted in reductions in liver mass that coincided with severe inflammation, histological damage, apoptosis, and induced gene expression of immature liver genes, immune responses, and cell-cycle regulation (191). Inhibition of hepatic CCR4–NOT activity by deletion of the deadenylase catalytic subunit CNOT6L led to increased mRNA expression of *Fgf21*, a hormone secreted mainly from the liver and involved in systemic glycemic control, due to inactivation of deadenylation. Expression of *Fgf21* resulted in increased serum FGF21 levels and improvements of diet-induced metabolic conditions and energy expenditure (192). These findings suggest that the CCR4-NOT complex-mediated mRNA decay mechanism is critical for proper liver function and systemic metabolic regulation, thus modulating the systemic metabolic-energy homeostasis and describing the strong association between post-transcriptional gene silencing and metabolism (192).

In addition to the role of CCR4-NOT complex in the liver, this complex apparently plays a vital regulatory role controlling adipocyte differentiation and functions. Adipocyte-specific Cnot3 conditional disruption results in lipodystrophy with hyperinsulinemia, hyperglycemia, insulin resistance, and glucose intolerance (193). Similar to the case of liver-specific Cnot3 conditional deletion, robust inflammation with activated macrophages was observed in the adipose tissue of Cnot3-deficiency, suggesting that deregulation of deadenylation causes stress and inflammatory responses. Cnot7, together with the members of Tob/BTG protein family, transducer of ERBB2 (Tob), regulates mRNA expression of uncoupling protein 1 (*Ucp1*), the main driver of non-shivering thermogenesis, in inguinal white adipose tissue (iWAT) (194). The Tob/BTG protein family is implicated in the regulation of mRNA decay via binding to the CCR4-NOT complex through direct interaction with Cnot7 (195–197). Tob interacts with BRF, the TTP family of ARE-recognizing protein, at the AU-rich region in the 3′-UTR of *Ucp1* mRNA, resulting in recruitment of Cnot7 deadenylase to *Ucp1* for its destabilization. Therefore, the absence of Cnot7 or Tob results in higher levels of *Ucp1* mRNA in iWAT. The increased *Ucp1* appears to protect from diet-induced obesity and associated complications in HFD-fed mice lacking Cnot7 and/or Tob deficiency (194). Conversely, Tob2, a member of the Tob/BTG protein family that interacts with Cnot7, inhibits adipogenesis, and its deficiency in mice induces increased adiposity. Tob2 blocks BMP2-induced Smad1/5 phosphorylation by directly interacting with Smad6, which sequentially suppresses mRNA expression of *Ppar*γ*-2*, a critical regulator of adipogenesis, although involvement of Tob2 in the regulation of *Ppar*γ*-2* mRNA stability remains elusive (198). These findings indicate that the CCR4-NOT complex-mediated post-transcriptional dysregulation of selective metabolic genes, and possibly global protein translation, are strongly associated with, and contribute to, the development of obesity and obesity-related disorders.

### Therapeutic Implications of Regulating the RNA-RBP Interaction

The complete understanding of the pathophysiology of obesity and the role of RNA-RBPs within the context of disease are still under investigation. However, there has been emerging *in vitro, ex vivo*, and *in vivo* experimental data highlighting potential underlying mechanisms of RNA-RBPs that might be crucial to answering important questions during the study of immune-metabolic dysregulation. As previously described, RNA-RBPs are considered novel players essential for the association between abnormal immune system response, posttranscriptional gene regulation, and metabolic dysfunction both at the tissue-specific and systemic scale. Clearly, it could be hypothesized that specific pharmacological inhibitors against particular RNA-RBPs would restore normal insulin signaling, glucose and lipid metabolism, and ameliorate inflammation and fibrosis of metabolic tissues, such as is observed in obesity-induced steatohepatitis. As a proof of principle, small chemical inhibitors were tested to block the activity of PKR, such as imoxin and 2-aminopurine (199, 200), and when used under immune-metabolic stress conditions resulted in improvements in immune-metabolic phenotypes in a mouse model (156). Additionally, new chemical agents are being developed that antagonize the miRNA-Ago2 protein complex, including anti-miRNA–Ago2 and tiny locked nucleic acids (LNAs), by binding to the active site of Ago2 protein and seed region of miRNAs, thereby resulting in functional inhibition of Ago2-mediated miRNAs (201–203). It is further reported that inhibitors of miRNA binding to Ago2 (miRISC loading inhibitors) were used in experimental studies which mimic Ago2 small hairpin RNA, although these have not been validated in clinical settings (204, 205). Collectively, these findings and efforts targeting specific RNA-RBP interactions may be useful toward developing a novel class of drugs designed to treat obesity-associated metabolic disorders.

Within the above context, pharmacological inhibition of upregulated miRNAs that contribute to the onset and propagation of obesity toward type 2 diabetes could potentially be employed as an alternative medical therapy, in which external artificial agents will be administered to counteract the miRNAs-induced molecular defects in obesity. For example, antisense oligonucleotides (AMOs) have been designed that bind to target miRNA and inhibit the function of miRNA by preventing its binding to target mRNA, followed by sequestration and/or RNase-mediated degradation. Currently, pharmaceutical laboratories have produced several generations of AMOs in order to improve the pharmacokinetic parameters with less toxicity by engineering AMOs containing other modifications, such as 2′-fluoro and/or 2′Omethoxyethyl, as well as 2′-O-methyl modifications, phosphorothioate bonds, and a 3′ end cholesterol tail seen in the first AMO generation (206–208). By taking advantage of the approach, it was demonstrated that specific miRNAs, such as miR-802, play a vital role in obesity-related hepatic insulin insensitivity and when reduced in mice by the AMOs they regained normal metabolic function (171). In a clinical setting, oligonucleotide inhibitors of liver-specific miR-122, RG-101 and Miravirsen, were used for clinical trials to treat patients with Hepatitis C virus (HCV) by targeting the 5′ end of miR-122 to diminish viral replication with certain results, thereby indicating the safety and effectiveness of the drug (209–212). This trial gives hope for the development of a new generation of drugs targeting specific RNAs.

Even in the presence of potentially effective, though limited, therapeutics targeting specific RNAs, further studies are required to elucidate the molecular mechanisms underlying the development of immune-metabolic disorders. Nonetheless, there is clear evidence that RNA-RBP networks play a critical role in the regulation of tissue-specific and/or whole-body glucose metabolism and inflammation, and that novel approaches targeting RNA-RBPs network in the setting of metabolic disorders will facilitate clinical research toward the identification of effective medical therapies for treating obesity and its associated metabolic diseases.

## Summary

Knowledge of the role of RNA networks associated with RNA modifications and RNA-RBP interactions in inflammatory and metabolic regulation in obesity is limited. However, accumulating evidence has shed light on how RNA networks dynamically affect metabolic, developmental, and inflammatory mechanisms in the pathogenesis of obesity-associated disorders. Indeed, functional changes of additional RBPs, including but not limited to LIN28A, IGF2BP2/IMP2, and HuR, are linked to the development of metabolic diseases (213–215). RNA methylation influences and fate maps RNA toward translation, degradation, or even sequestration inside cellular compartments. Therefore, reversible RNA modification, particularly RNA methylation, represents a new epigenetic marker, similar to reversible DNA modifications, that provides clues to adaptive cellular responses associated with metabolic changes in response to distinct exogenous or endogenous stress stimuli including excessive nutrients, toxins, and microbial infections (216, 217). RNA modifications could affect RNA-RBP networks and emerging biological events, including RNA export and transport, RNA cleavage, maturation, and stability, as well as functional changes in RBPs (218). Unveiling these epigenetic regulatory roles of the RNA networks is important to clarify the pathogenesis of chronic diseases including, but not limited to, obesity, cardiovascular, aging, and autoimmune diseases, where the exact molecular mechanisms are still ill defined.

While RNA modifications are generally considered a fine-tuning process of cellular homeostasis, prolonged external cues, including chronic HFD-feeding, may gradually accumulate erroneous RNA modifications that drive the cell fate toward chronic and low-grade inflammatory conditions which are well-observed cellular features in obesity. This concept is supported by evidence that several inflammatory RBPs, including TLR3, TLR7, and PKR, are involved in the induction of obesity-associated chronic inflammation. As mRNA methylation modification could influence post-transcriptional gene silencing and control targeted gene expression, e.g., through affecting interactions with mRNA/miRNA/Ago proteins complex, there may be specific types and modifications of RNAs that activate metabolic and inflammatory programs in the pathogenesis of obesity.

Current technical advances have allowed us to investigate quantitative changes in many types of RNA species; either coding or non-coding RNA levels, and methylation status (219–221). However, the challenge is to precisely analyze the type of modification, the degree of modifications, and to predict their biological significance, particularly the effect of these changes on the RNA's secondary structure, localization, and interactions with specific RBPs. These important insights would then lead to the development of novel therapeutics to change RNA-RBP interactions in obesity and T2D. Nonetheless, to investigate the components that manage RNA specificity, regulatory mechanisms, and functions of RBPs, it is important to prioritize methods that characterize direct endogenous protein-RNA interactions. In the past decade, several RIP methods, including cross-linking and immunoprecipitation (CLIP) and methylation individual-nucleotide-resolution CLIP (miCLIP), have been developed to determine the *in vivo* RNA targets of RBPs (221–223). Utilization of RBPs known to be involved in inflammatory and metabolic diseases to identify the “pathological” RNAs by RIP would provide unique approaches for better understanding the molecular mechanisms of chronic inflammatory diseases. Such efforts might pave the way for novel therapeutic and pharmacological targets and/or interventions for combating obesity-induced sequelae.

## Author Contributions

All authors listed have made a substantial, direct and intellectual contribution to the work, and approved it for final version.

### Conflict of Interest Statement

The authors declare that the research was conducted in the absence of any commercial or financial relationships that could be construed as a potential conflict of interest.
